# A Rare Case of Wolfram Syndrome in a 27-Year-Old Male From Nepal

**DOI:** 10.7759/cureus.95410

**Published:** 2025-10-25

**Authors:** Tej P Shah, Richard Sidlow, Prem K Sah

**Affiliations:** 1 Diabetes and Endocrinology, National Academy of Medical Sciences, Kathmandu, NPL; 2 Medical Genetics and Metabolism, Valley Children's Hospital, Madera, USA; 3 Medicine, Sukraraj Tropical and Infectious Disease Hospital, Kathmandu, NPL

**Keywords:** diabetes mellitus, genetic disease, optic atrophy, sensorineural (sn) hearing loss, type 1 and type 2 diabetes mellitus, wolfram like syndrome, wolfram syndrome, wolfram syndrome 1 (wfs1), wolfram syndrome 2 (wfs2)

## Abstract

Wolfram syndrome is a rare autosomal recessive disorder characterized by diabetes mellitus, optic atrophy, and progressive neurodegeneration, often summarized by the acronym DIDMOAD (diabetes insipidus, diabetes mellitus, optic atrophy, and deafness). We report a 27-year-old male with a history of diabetes mellitus, progressive visual loss leading to blindness, and bilateral sensorineural hearing loss. His family history was notable for diabetes-related mortalities and visual impairment in multiple members of the family. Clinical evaluation showed uncontrolled blood glucose level, optic atrophy, and high-frequency sensorineural hearing loss. A clinical diagnosis of Wolfram syndrome was made using the Euro-Wolfram, Alström, and Bardet-Biedl (WABB) criteria. Wolfram syndrome should be suspected in young patients with early-onset diabetes mellitus and visual or hearing impairment. This case report highlights the role of timely multidisciplinary management in preventing disease-related complications.

## Introduction

Wolfram syndrome is an autosomal recessive disorder characterized by early-onset diabetes mellitus, optic atrophy, and progressive neurodegeneration, including deafness. Wolfram and Wegener first described this disease in 1938; four of the eight siblings presented with both diabetes and optic atrophy [1,2]. Other abnormalities associated with the Wolfram syndrome include genitourinary abnormalities, cerebellar ataxia, brainstem dysfunction, and epilepsy [3].

The prevalence of Wolfram syndrome is approximately one in 770,000 in the United Kingdom and one in 100,000 in North America [4]. Genetic testing distinguishes optic atrophy from other causes of juvenile optic atrophy [5].

We are reporting a case of Wolfram Syndrome in a young adult male from Nepal, diagnosed clinically using the Euro-Wolfram, Alström, and Bardet-Biedl (WABB) criteria [6].

## Case presentation

A 27-year-old man presented with a non-healing ulcer on his left foot for two months, following trauma. He had been diagnosed with type 2 diabetes mellitus (T2DM) two years earlier when he developed polyuria and polydipsia. At that time, he was started on oral anti-diabetic drugs and has had no history of diabetes-related emergencies until the current presentation.

The patient reported progressive bilateral vision loss since early childhood, resulting in complete blindness. He didn't complain of any history of decreased hearing, polyuria suggestive of diabetes insipidus, neurological deficits, or genitourinary symptoms. 

**Figure 1:**
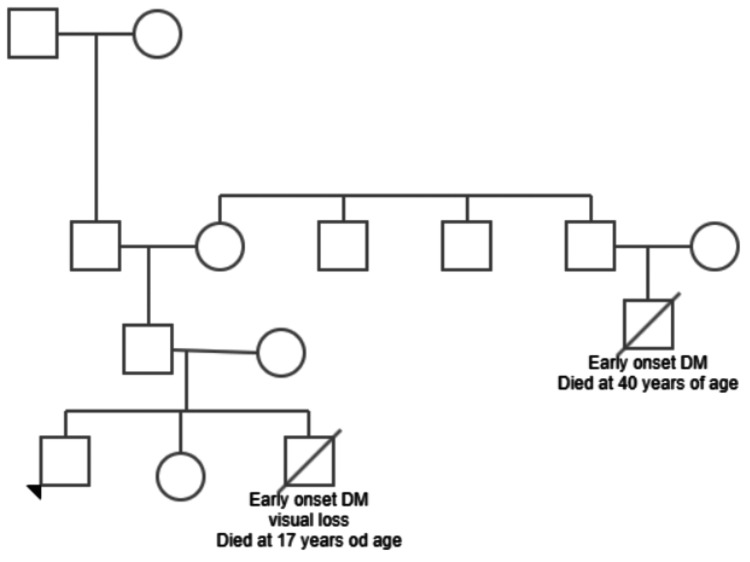
Family pedigree. The pedigree demonstrates that the proband (the patient being reported, indicated by the arrowhead), his brother, and his paternal granduncle were affected with early-onset diabetes mellitus (DM), with visual impairment noted in the patient and his brother. The granduncle died at 40 years of age due to complications of diabetes, while the patient's brother died at 17 years of age following early-onset diabetes and visual loss. The inheritance pattern in this family suggests a possible genetic etiology.

His family history is significant for the death of a sibling (elder brother) at the age of 17 years due to diabetes-related complications, and he also had visual impairment. His granduncle had died in his early adulthood with similar features. There is no history of consanguinity in the parents. The patient's family pedigree is shown in Figure 1.

He was admitted to our hospital for optimization of blood glucose levels. Initial investigations revealed uncontrolled hyperglycemia, with fasting blood glucose of 308 mg/dL, postprandial glucose of 403 mg/dL, and HbA1c of 16%. He also had significant dyslipidemia, with total cholesterol of 342 mg/dL, triglycerides of 456 mg/dL, and reduced high-density lipoprotein (HDL) cholesterol at 41 mg/dL. Serum creatinine was mildly elevated at 1.4 mg/dL, while serum electrolytes and urea were within normal limits. Urinalysis showed 4+ glycosuria and 1+ albuminuria.

**Figure 2:**
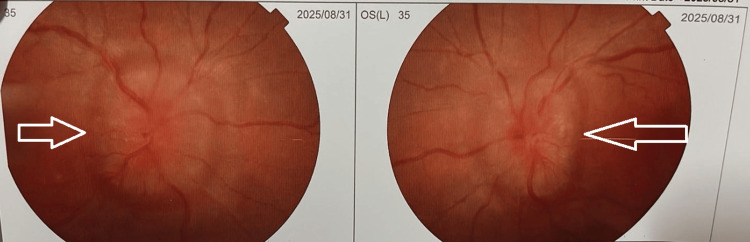
Ophthalmoscopy view of bilateral optic atrophy (arrows). On fundoscopic examination, both optic discs appeared pale with clearly defined margins, consistent with optic atrophy. The retinal vessels were mildly attenuated, and the surrounding retina showed no evidence of hemorrhages, exudates, or edema.

On further investigation, pure tone audiometry (PTA) showed bilateral sensorineural hearing loss of 41 dB and 40 dB in the right and left ears, respectively, with high-frequency loss. On ophthalmoscopy examination, he had bilateral optic atrophy on fundoscopy, as shown in Figure 2. There was no cerebellar ataxia, and deep tendon reflexes were normal. Ultrasound of the abdomen and pelvis showed no renal abnormalities.

The patient was switched to a basal-bolus insulin regimen for optimal glycemic control. Regular wound care and blood sugar control led to complete healing of the foot ulcer. He is currently under regular follow-up in our hospital.

The clinical diagnosis of Wolfram syndrome was made based on the presence of two major diagnostic criteria (diabetes mellitus and optic atrophy) along with sensorineural hearing loss. The pedigree pattern was consistent with a hereditary pattern of inheritance.

## Discussion

The acronym DIDMOAD (diabetes insipidus, diabetes mellitus, optic atrophy, and deafness) is widely used to describe the classical clinical spectrum of the Wolfram syndrome [3].

Diabetic mellitus occurs in 98% cases of Wolfram syndrome, but may not be the first presenting feature in 20% cases. It is non-autoimmune, insulin-deficient, and non-human leukocyte antigen (HLA)-linked. No antibodies against insulin are found [2,3,7]. Our patient was initially diagnosed with type 2 diabetes mellitus and was treated with oral anti-diabetic drugs; his blood sugar was not under control, as evidenced by significantly high HbA1c. The patient was subsequently started on insulin and has achieved good glycemic control till now.

Optic atrophy is the second most common sign of Wolfram syndrome, occurring in 82% cases. It presents as painless progressive bilateral vision diminution. The mean age of onset in Wolfram syndrome is 11 years [2,7]. In our case, vision impairment began in early childhood and progressed to complete blindness.

Sensorineural hearing loss occurs in 70% cases and initially affects high frequencies, followed by relatively lower frequencies [2,7]. Our patient’s audiometric evaluation revealed mild bilateral sensorineural hearing loss, predominantly affecting high frequencies.

Other manifestations, such as central diabetes insipidus (in 38% of cases), urinary tract abnormalities (in 19% of cases), and neurological symptoms such as cerebellar ataxia, central apnea, anosmia, and peripheral neuropathy, typically present later, around age 30. Hyponatremia and hypogonadism due to pituitary insufficiency have also been reported [2,7,8]. Our patient did not have these features at the time of presentation.

Wolfram syndrome is considered a prototype of endoplasmic reticulum (ER) diseases; the ER is a membrane-bound organelle crucial for protein folding, calcium homeostasis, redox balance, lipid and steroid synthesis, intracellular signaling, and regulation of programmed cell death [9,10]. In Wolfram syndrome, pancreatic β-cells and neuronal cells undergo selective degeneration due to mutations in the WFS1 gene. This gene encodes wolframin, a transmembrane protein localized to the endoplasmic reticulum (ER) membrane, where it plays a crucial role in maintaining ER homeostasis and cellular calcium balance [5,11]. A small portion of patients have the WFS2 (CISD2) gene mutation. WFS2 also encodes a protein localized to the ER. In patients with WFS2 mutations, diabetes mellitus and hearing impairment are reported. They differ from patients carrying the WFS1 mutation who do not have diabetes insipidus [12,13].

The main cause of mortality in Wolfram syndrome is central respiratory failure due to brain stem atrophy or renal failure due to infection. The prognosis is poor, with a mean age of death of 30-40 years [2,14].

A limitation of this case is the lack of genetic testing, which was not feasible due to its unavailability at the local level and the patient’s financial constraints. Without genetic confirmation, it is not possible to differentiate between Wolfram syndrome type 1 (WFS1 gene mutation), Wolfram syndrome type 2 (WFS2 gene mutation), Wolfram-like syndrome (heterozygous WFS1 mutation), and maternally inherited diabetes and deafness (MIDD). Although the patient is unmarried and does not plan to have children, and thus a confirmed genetic diagnosis would not alter his clinical management, it would still be valuable for genetic counseling and risk assessment in other family members.

## Conclusions

Wolfram syndrome is a rare genetic disorder that should be considered in the differential diagnosis for patients presenting with early-onset diabetes mellitus accompanied by auditory and visual impairment. The diagnosis and management of this condition necessitate a multidisciplinary approach to address its diverse manifestations. This case underscores the importance of maintaining a high index of clinical suspicion and utilizing established diagnostic criteria, such as the Euro-Wolfram, Alström, and Bardet-Biedl (WABB), for identifying rare syndromes in resource-limited settings like Nepal, even without genetic confirmation. Ultimately, this report aims to enhance recognition and facilitate timely intervention to mitigate complications among patients in this region.
